# Association Between Pre-existing Comorbidities and Post-admission Complications and Outcomes in Older Trauma Patients Using a Nationwide Trauma Registry Database in Japan

**DOI:** 10.7759/cureus.104871

**Published:** 2026-03-08

**Authors:** Sayaka Noguchi, Daizoh Saitoh, Hideharu Tanaka

**Affiliations:** 1 Graduate School of Emergency Medical System, Kokushikan University, Tokyo, JPN; 2 Department of Emergency Medical Science, Kyoto Tachibana University, Kyoto, JPN

**Keywords:** charlson comorbidity index, cox regression analysis, elderly trauma, in-hospital mortality, japan trauma data bank

## Abstract

Objective

This study aims to identify preexisting comorbidities associated with increased in-hospital mortality among older trauma patients and to examine whether specific comorbidities are linked to distinct clinical outcomes characterized by severe post-admission complications.

Methods

This study included 77,336 patients aged ≥65 years with blunt or penetrating trauma who were admitted to participating facilities of the Japan Trauma Data Bank between January 2019 and December 2023. Patients with out-of-hospital cardiac arrest (OHCA) were excluded. Cox proportional hazards regression was performed using age, sex, trauma type (blunt or penetrating), presence or absence of severe injury in nine Abbreviated Injury Scale (AIS) regions, trauma severity scores (Revised Trauma Score and Injury Severity Score), and individual components of the Charlson Comorbidity Index (CCI) as explanatory variables. Survival time was defined as the dependent variable, and in-hospital outcome was treated as the event. Among CCI items significantly associated with mortality, hierarchical cluster analysis using Ward’s method was conducted to identify the patterns of preexisting comorbidities. Furthermore, Fisher's exact test was used to evaluate associations between individual CCI items and postoperative in-hospital complications related to mortality, including central nervous system, cardiovascular, gastrointestinal, and urinary tract complications; acute renal failure; abdominal compartment syndrome; pneumonia; disseminated intravascular coagulation (DIC); sepsis; and infections.

Results

In the Cox proportional hazards analysis, the CCI components with the highest hazard ratios (HR) for hospital mortality were metastatic solid tumors (HR: 2.926), moderate or severe liver disease (HR: 2.860), moderate or severe renal impairment (HR: 1.888), leukemia (HR: 1.782), congestive heart failure (HR: 1.667), mild liver disease (HR: 1.474), connective tissue disease (HR: 1.417), and chronic lung disease (HR: 1.365) (all *p*<0.05). Most comorbidities show broad, non-specific associations across multiple complication categories, while connective tissue disease showed a comparatively narrower pattern confined to central nervous system and urinary system complications. Moreover, hierarchical cluster analysis showed that connective tissue disease formed the most distinct cluster among the eight mortality-associated CCI components.

Conclusion

In-hospital mortality among older trauma patients was associated not only with trauma severity but also with eight preexisting comorbidities included in the CCI. Pre-existing cardiac, pulmonary, hepatic, or renal dysfunction, as well as malignancy, were independent risk factors for mortality following blunt or penetrating trauma. Among these comorbidities, connective tissue disease was associated with a unique pattern of complications following hospitalization, suggesting a different occurrence of complications compared with other preexisting conditions in older trauma patients.

## Introduction

As the aging population increases, the number of older trauma patients increases annually, making trauma one of the leading causes of death among older adults [1]. In aged societies such as Japan, where individuals aged ≥65 years account for approximately 30% of the population, the prevention and appropriate treatment of trauma in older adults represent urgent clinical challenges. Older adults are more likely to experience severe outcomes even after minor injuries because of age-related declines in physiological reserve capacity, increased fragility, and the presence of multiple comorbidities [2]. Previous studies have demonstrated that prognosis in older trauma patients is influenced not only by injury mechanism and severity but also by medical history, comorbid conditions, and post-injury complications [3]. Among available measures, the Charlson Comorbidity Index (CCI) [4] quantifies comorbidity burden across 19 disease categories and is widely used for prognostic assessment; however, it was not designed specifically for trauma-related comorbidities [3]. Nevertheless, higher total CCI scores have been associated with increased hospitalization and mortality rates, indicating its potential utility for risk stratification in older trauma patients [5].

Many existing studies, however, include all age groups, and mortality risk factors specific to older adults. Parreira et al. reported that advanced age alone confers high risk, regardless of medical history, injury location, or injury mechanism [6]. These findings suggest age-related pathophysiological differences between younger and older trauma patients and indicate that older trauma patients require more detailed clinical evaluation. Furthermore, trauma severity scores, including the Injury Severity Score (ISS) [7], Revised Trauma Score (RTS) [8], and Trauma and Injury Severity Score (TRISS) [9], have reduced prognostic accuracy in older patients compared with younger individuals [1]. In head trauma, even injuries classified as mild may progress to severe outcomes, and age has been identified as an independent prognostic factor. Older patients exhibit higher mortality rates and poorer functional outcomes than their younger counterparts with comparable injury severity [10]. Moreover, age-related bone atrophy and degenerative changes may obscure injuries on imaging, increasing the risk of delayed diagnosis [11]. Comorbidities and activities of daily living (ADL) status also significantly influence the likelihood of interfacility transfer or return home after hospitalization [12]. Therefore, accurate identification of comorbidities in older patients is essential not only for survival but also for improving overall prognosis. Beyond comorbidity burden and pre-injury functional status, frailty, reflecting reduced physiologic reserve and stressor tolerance, has been consistently associated with higher mortality, complications, prolonged hospitalization, and unfavorable discharge disposition in older trauma patients, and may complement conventional injury severity metrics [13].

This study aimed to identify preexisting comorbidities associated with increased mortality risk among older trauma patients aged ≥65 years and to examine characteristic comorbidities that influence outcomes, focusing on post-admission complications. Using nationwide data from the Japan Trauma Data Bank (JTDB) [14,15,16], this retrospective exploratory study sought to identify high-risk preexisting comorbidities that may inform prehospital assessment. The novelty of this paper are the following: (a) evaluation of individual CCI components rather than the overall CCI score; (b) usage of the big trauma registry JTDB from 2019-2023; (c) combining survival analysis with exploratory clustering; and (d) attempting to link comorbidity patterns with complication profiles.

## Materials and methods

Study design

This retrospective cohort study used data from the new JTDB database [14-16]. The database is a nationwide trauma registry established by the Japanese Society for Trauma Surgery and the Japanese Association for Acute Medicine to improve the quality of trauma care in Japan. The number of participating hospitals has increased annually, with 303 facilities contributing data as of April 2023. The JTDB transitioned from its former system to a new system in 2019. This study utilized case data recorded in the new JTDB system for patients admitted to participating facilities between 2019 and 2023. The JTDB database used in this study was sent as anonymized data in February 2025 to 93 facilities affiliated with the Japan Trauma Research and Care Organization (including one facility, the Graduate School of Emergency Medical Systems at Kokushikan University) for the purpose of conducting trauma epidemiological research [17].

Ethical declaration

This study was conducted in accordance with the principles of the Declaration of Helsinki. Ethical approval was obtained from the Ethics Committee of the Department of Sports Medicine, Faculty of Physical Education, Kokushikan University (Approval Number: 25040).

Study endpoints

The primary endpoint was in-hospital mortality among older trauma patients aged ≥65 years. The secondary endpoint was to identify the preexisting comorbidities associated with in-hospital mortality, particularly those linked to distinct post-admission complication patterns; that is, clustering and complication analyses were conducted as secondary/exploratory.

Participants

A total of 176,054 hospitalized cases recorded in the new JTDB system between January 1, 2019, and December 31, 2023, were initially identified. Cases except blunt or penetrating trauma were excluded, as were cases involving cardiac arrest at the scene or upon hospital arrival. Patients younger than 65 years of age and those of unknown age were further excluded from the analysis in this study. These cases were classified into in-hospital survivors and in-hospital deaths.

Data used

From the variables available in the new JTDB system [15-17], the following data were extracted: age, sex, injury mechanism, systolic blood pressure (sBP) upon hospital arrival, Glasgow Coma Scale (GCS) score upon hospital arrival [18], Injury Severity Score (ISS) [7], RTS on hospital arrival [8], maximum AIS score across nine body regions based on AIS 2005 update 2008 [19] (hereafter AIS 2008), total CCI score, and individual comorbidity components, post-admission complications, and discharge outcome (survival or death).

The AIS score (the number after the dot) is an indicator for assessing the severity of injury in each anatomical region. AIS.1 indicates minor injury, AIS.2 moderate injury, AIS.3 severe injury, AIS.4 critical injury, AIS.5 near-fatal injury, and AIS.6 instant death. Therefore, cases with an AIS score of three or higher are classified as life-threatening severe injuries. Additionally, region one denotes the head, two the face, three the neck, four the chest, five the abdomen, six the spine, seven the upper limbs, eight the lower limbs, and nine the skin. Cases with the highest injury severity score of three or higher (AIS regions 1-9, max score ≥3) within each region are classified as severe cases. The proportion of severe cases among all cases is shown separately for each region. Furthermore, the ISS was calculated by selecting the maximum AIS score from three different body regions among the six regions: head and neck, face, chest, abdomen (including pelvic organs), extremities (including pelvis), and skin surface, squaring each score, and summing them. RTS was calculated by coding and weighting three admission items: Glasgow Coma Scale, systolic blood pressure, and respiratory rate. The CCI is an index that evaluates the total score by weighting 19 predefined comorbidities. However, in this paper, each case was evaluated based on whether or not it had each of the 19 comorbidities.

Statistical methods

The analysis of the primary endpoint, in-hospital mortality, was performed using Cox proportional hazards regression analysis. For the secondary endpoints, the association between preexisting comorbidities and postoperative complications, and the clustering of these complications, Fisher's exact test and cluster hierarchy analysis were employed.

Baseline characteristics, ISS, RTS, and CCI, were summarized by discharge outcome for older patients with blunt or penetrating trauma. Group comparisons according to discharge outcome were conducted for each CCI component [4]. Cox proportional hazards regression analysis was then performed, with survival time as the dependent variable and discharge outcome as the event. Explanatory variables included age, sex, trauma type (blunt or penetrating), RTS, ISS, presence of severe injury (maximum AIS score ≥3 in any of the AIS 1-9 regions), and individual CCI item.

For the CCI item significantly associated with mortality in the Cox regression analysis, hierarchical cluster analysis using Ward’s method was performed. Variables included in the clustering were age, sex, ISS, RTS, discharge outcome, and length of hospital stay, to identify pre-existing comorbidities associated with different post-admission clinical courses.

Furthermore, associations between each mortality-associated CCI item and postoperative complications were examined using the full cohort. Complications analyzed included central nervous system, cardiovascular, gastrointestinal, and urinary tract complications, acute renal failure, abdominal compartment syndrome, pneumonia, disseminated intravascular coagulation (DIC), sepsis, and infection.

Categorical variables were presented as counts and percentages. Welch’s t-test was used to compare two continuous groups, and Fisher’s exact test for categorical variables. The results of multiple regression analysis were reported as partial regression coefficients, standard errors, standardized regression coefficients, odds ratios, p-values, and 95% confidence intervals (95%CI). Cox regression results were reported as standardized regression coefficients (SRC), hazard ratios (HR), p-values, and 95%CI. In the hierarchical clustering analysis, mortality rates were expressed as percentages, and time to death was recorded to one decimal place. Statistical significance was set at p<0.05. Statistical analyses were performed using JMP Pro version 15. 0. 0 (SAS Institute, Cary, NC, USA) and SPSS version 29 (IBM Corp., Armonk, NY, USA).

## Results

**Figure 1:**
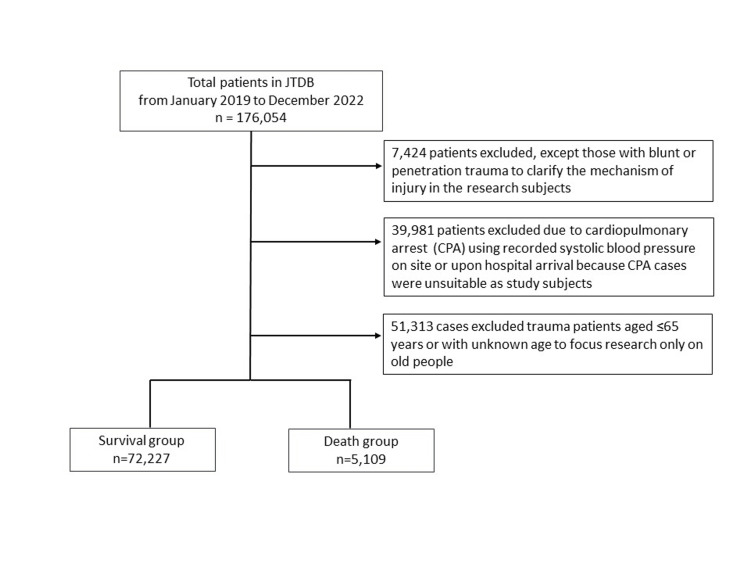
Study flow diagram of the included patients.

Figure 1 presents the flowchart of case selection for the 77,336 patients included in this study. Of the 176,054 cases registered in the new JTDB database released in 2023, 7,424 cases involving burns, blast injuries, or unknown trauma mechanisms were excluded because they did not involve blunt or penetrating trauma. Based on sBP recorded at the scene and upon hospital arrival, 39,381 cases of out-of-hospital cardiac arrest (OHCA) at emergency crew contact and in-hospital cardiac arrest (IHCA) were further excluded. Additionally, 51,313 cases involving patients aged <65 years or with unknown age were excluded. The final study population comprised 77,336 cases, including 72,227 in-hospital survivors and 5,109 in-hospital deaths.

Tables 1-3 summarize the comparisons of patient characteristics between the in-hospital mortality and survival groups. Compared with survivors, patients in the mortality group were significantly older (81.9±8.0 years, p<0.001; Table 2) and more frequently men (64.6%, p<0.001; Table 1). Blunt trauma predominated in both groups (Table 1); however, its proportion was significantly higher in the mortality group than in the survival group (99.1%, p<0.001). As shown in Table 2, trauma severity indices differed markedly between groups: the mortality group had a significantly lower RTS (6.03±1.67, p<0.001) and a significantly higher ISS (22.3±12.2, p<0.001), indicating greater injury severity. Furthermore, the proportion of cases with AIS≥3 was significantly higher in the mortality group for the head, face, chest, and abdomen (all p<0.001), whereas it was significantly lower for the spine, upper extremities, lower extremities, and skin/subcutaneous tissue (all p<0. 001) (Table 1). Regarding pre-existing comorbidities, myocardial infarction (4.5%), congestive heart failure (6.2%), peripheral vascular disease (1.3%), chronic lung disease (4.3%), mild liver disease (2.0%), diabetes with organ damage (2.3%), moderate or severe renal impairment (5.2%), malignancy (7.1%), leukemia (0.6%), moderate or severe liver disease (0.6%), and metastatic malignancy (2.3%) were all significantly more frequent in the mortality group than in the survival group (Table 3).

**Table 1:** Demographic characteristics, trauma type, and AIS body region severity p-value calculated by Fisher’s exact test. AIS: Abbreviated Injury Scale.

Variable	Death: n (%)	Survival: n (%)	p value	Odds ratio
Sex (Male)	3,288 (64.6)	33,908 (47.2)	<0.001	2.046
(Female)	1,798 (35.4)	37,935 (52.8)	<0.001	0.489
Trauma type (Blunt)	5,062 (99.1)	70,928 (98.2)	<0.001	1.972
(Penetrating)	47 (0.9)	1,299 (1.8)	<0.001	0.507
AIS body region 1 max score ≧ 3	3,346 (66.9)	14,837 (21.0)	<0.001	7.611
AIS body region 2 max score ≧ 3	25 (0.5)	145 (0.2)	<0.001	2.455
AIS body region 3 max score ≧ 3	12 (0.2)	187 (0.3)	0.886	0.907
AIS body region 4 max score ≧ 3	1,131 (22.6)	11,586 (16.4)	<0.001	1.491
AIS body region 5 max score ≧ 3	256 (5.1)	1,537 (2.2)	<0.001	2.428
AIS body region 6 max score ≧ 3	464 (9.3)	8,345 (11.8)	<0.001	0.764
AIS body region 7 max score ≧ 3	22 (0.4)	635 (0.9)	<0.001	0.488
AIS body region 8 max score ≧ 3	1,068 (21.4)	29,835 (42.2)	<0.001	0.372
AIS body region 9 max score ≧ 3	35 (0.7)	187 (0.3)	<0.001	2.658

**Table 2:** Age and trauma severity measures stratified by in-hospital outcome (n=77,336) p-value calculated by the Welch’s t-test, SD: standard deviation.

Variable	Death: Mean±SD	Survival: Mean±SD	p	Hedges' g
Age	81.9±8.0	80.5±8.4	<0.001	0.176
Revised Trauma Score	6.03±1.67	7.63±0.62	<0.001	2.168
Injury Severity Score	22.3±12.2	12.1±7.4	<0.001	1.298

**Table 3:** Comparisons of Charlson Comorbidity Index items between two groups (n=77,336) p-value calculated  by Fisher’s exact test.

Variable	Missing (n)	Death: n (%)	Survival: n (%)	p value	Odds ratio
Myocardial infarct	30	232 (4.5)	2,366 (3.3)	<0.001	1.404
Congestive heart failure	30	319 (6.2)	2,810 (3.9)	<0.001	1.644
Peripheral vascular disease	30	68 (1.3)	710 (1.0)	0.018	1.358
Cerebrovascular disease	30	470 (9.2)	6,515 (9.0)	0.67	1.021
Dementia	30	709 (13.9)	10,209 (14.1)	0.617	0.978
Chronic pulmonary disease	30	220 (4.3)	2,586 (3.6)	0.008	1.211
Connective tissue disease	30	67 (1.3)	1,032 (1.4)	0.537	0.916
Ulcer disease	29	103 (2.0)	1,371 (1.9)	0.564	1.063
Mild liver disease	30	103 (2.0)	1,105 (1.5)	0.007	1.324
Diabetes	29	806 (15.8)	12,027 (16.7)	0.103	0.937
Diabetes with end organ damage	30	115 (2.3)	1,139 (1.6)	<0.001	1.437
Moderate or severe renal damage	30	266 (5.2)	2,148 (3.0)	<0.001	1.791
Hemiplegia	30	87 (1.7)	1,320 (1.8)	0.548	0.93
Any tumor	30	362 (7.1)	4,511 (6.2)	0.018	1.144
Leukemia	30	30 (0.6)	140 (0.2)	<0.001	3.04
Lymphoma	30	36 (0.7)	403 (0.6)	0.175	1.264
Moderate or severe liver disease	30	30 (0.6)	155 (0.2)	<0.001	2.745
Metastatic solid tumor	30	119 (2.3)	681 (0.9)	<0.001	2.504
AIDS	29	2 (0.04)	24 (0.03)	0.689	1.178

Tables 4, 5 present the results of the Cox proportional hazards model. The Cox proportional hazards model was statistically significant (χ²=15268.066, df=33, p<0.001), indicating improved model fit over the null model (-2 log likelihood=66650.875). Age was significantly associated with in-hospital mortality, with an HR of 1.055 per one-year increase (95%CI: 1.050-1.060, p<0.001). Male sex was also significantly associated with increased mortality compared with female sex (HR 1.464, 95%CI: 1.367-1.575, p<0. 001). Trauma type (blunt vs penetrating) was not significantly associated with mortality. Among severity indices at admission, RTS was inversely associated with in-hospital mortality (HR 0.544, 95%CI: 0.531-0.556, p<0.001), whereas ISS was positively associated with in-hospital mortality (HR 1.034, 95%CI: 1.030-1.037, p<0.001). Regarding AIS severity, involvement of body region 1 or 5 with AIS ≥3 was significantly associated with increased in-hospital mortality, whereas involvement of regions 3, 4, 6, 7, or 8 was significantly associated with in-hospital survival.

**Table 4:** Association between CCI components and In-hospital mortality by Cox regression analysis (n=67,476) SRC: standardized regression coefficient, HR: hazard ratio, CI: confidence interval. AIS: Abbreviated Injury Scale.

Variable	SRC	p	HR	95%CI
Age	0.053	<0.001	1.055	1.050-1.060
Sex (male)	0.381	<0.001	1.464	1.360-1.575
Trauma type (blunt)	0.087	0.627	1.091	0.768-1.551
Revised Trauma Score	-0.61	<0.001	0.544	0.531-0.556
Injury Severity Score	0.033	<0.001	1.034	1.030-1.037
AIS body region 1 max score ≧ 3	0.748	<0.001	2.133	1.916-2.331
AIS body region 2 max score ≧ 3	-0.216	0.314	0.805	0.538-1.228
AIS body region 3 max score ≧ 3	-0.743	0.028	0.475	0.245-0.923
AIS body region 4 max score ≧ 3	-0.242	<0.001	0.785	0.716-0.861
AIS body region 5 max score ≧ 3	0.176	0.031	1.193	1.016-1.399
AIS body region 6 max score ≧ 3	-0.62	<0.001	0.538	0.468-0.618
AIS body region 7 max score ≧ 3	-0.801	0.002	0.449	0.269-0.748
AIS body region 8 max score ≧ 3	-0.592	<0.001	0.553	0.502-0.609
AIS body region 9 max score ≧ 3	-0.109	0.606	0.896	0.591-1.359
Myocardial infarct	-0.027	0.741	0.974	0.831-1.141
Congestive heart failure	0.511	<0.001	1.667	1.453-1.913
Peripheral vascular disease	0.103	0.527	1.108	0.806-1.523
Cerebrovascular disease	0.109	0.739	1.019	0.911-1.141
Dementia	0.044	0.381	1.045	0.947-1.155
Chronic pulmonary disease	0.311	<0.001	1.365	1.161-1.604
Connective tissue disease	0.349	0.014	1.417	1.073-1.871
Ulcer disease	0.049	0.693	1.05	0.825-1.337
Mild liver disease	0.388	0.001	1.474	1.168-1.859
Diabetes	0.015	0.75	1.015	0.927-1.111
Diabetes with end organ damage	0.068	0.555	1.071	0.854-1.343
Moderate or severe renal damage	0.636	<0.001	1.888	1.616-2.206
Hemiplegia	-0.142	0.289	0.867	0.666-1.129
Any tumor	0.066	0.315	1.068	0.939-1.214
Leukemia	0.578	0.009	1.782	1.157-2.744
Lymphoma	0.097	0.63	1.102	0.742-1.638
Moderate or severe liver disease	1.051	<0.001	2.86	1.934-4.228
Metastatic solid tumor	1.073	<0.001	2.926	2.368-3.615
AIDS	-8.341	0.146	0	0.000-0.001

**Table 5:** Profile of explanatory variables in Cox regression analysis (n=67,476; case NMV in multivariate analysis: 9860) NMV: Number of missing values, VIF: variance inflation factor. AIS: Abbreviated Injury Scale.

Variable	NMV	VIF
Observation days	3,245	-
In-hospital outcome	1	-
Age	0	1.237
Sex (male)	407	1.199
Trauma type (blunt)	0	1.072
Revised Trauma Score	1832	2.501
Injury Severity Score	5705	1.326
AIS body region 1 max score ≧ 3	1611	2.289
AIS body region 2 max score ≧ 3	1611	1.007
AIS body region 3 max score ≧ 3	1611	1.029
AIS body region 4 max score ≧ 3	1611	1.659
AIS body region 5 max score ≧ 3	1611	1.112
AIS body region 6 max score ≧ 3	1611	1.738
AIS body region 7 max score ≧ 3	1611	1.044
AIS body region 8 max score ≧ 3	1611	2.026
AIS body region 9 max score ≧ 3	1611	1.022
Myocardial infarct	30	1.019
Congestive heart failure	30	1.032
Peripheral vascular disease	30	1.01
Cerebrovascular disease	29	1.016
Dementia	30	1.115
Chronic pulmonary disease	30	1.009
Connective tissue disease	30	1.009
Ulcer disease	29	1.005
Mild liver disease	30	1.004
Diabetes	29	1.015
Diabetes with end organ damage	30	1.068
Moderate or severe renal damage	30	1.07
Hemiplegia	30	1.006
Any tumor	30	1.007
Leukemia	30	1.001
Lymphoma	30	1.002
Moderate or severe liver disease	30	1.003
Metastatic solid tumor	30	1.002
AIDS	29	1.001

**Figure 2:**
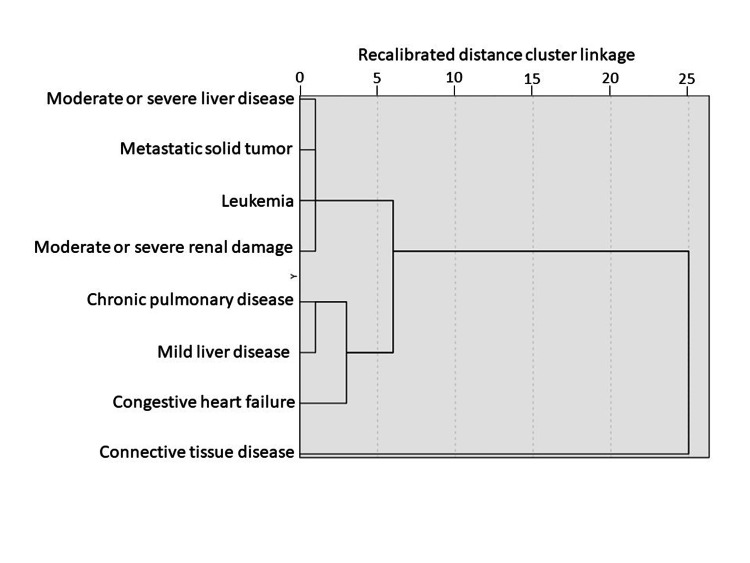
Dendrogram based on Ward's method using class cluster analysis.

Figure 2 illustrates the results of hierarchical cluster analysis using Ward’s method for the eight preexisting comorbidities significantly associated with in-hospital mortality, based on age, sex, ISS, RTS, mortality rate, and days to death (Table 6). Several distinct clusters were identified. Moderate or severe liver disease, metastatic solid tumors, leukemia, and moderate or severe renal impairment formed closely related clusters, whereas chronic lung disease, mild liver disease, and congestive heart failure formed a separate cluster. Notably, connective tissue disease was markedly isolated from the other pre-existing comorbidities.

**Table 6:** Profile of comorbidity before injury for class cluster analysis (n=77,336) RTS: Revised Trauma Score; ISS: Injury Severity Score.

Comorbidity before injury	Age	Male	ISS	RTS	Mortality	Days to death
	Mean±SD	n (%)	Mean±SD	Mean±SD	n (%)	Mean±SD
Congestive heart failure	85.4±7.7	1,313 (42.1)	11.5±6.5	7.58±0.72	319 (10.2)	24.2±31,2
Chronic pulmonary disease	81.6±8.0	1,474 (52.9)	11.9±7.3	7.60±0.69	220 (7.8)	24.2±22.9
Connective tissue disease	79.7±7.5	243 (22.2)	11.6±7.7	7.62±0.74	67 (6.1)	24.6±22.8
Mild liver disease	78.8±7.9	608 (50.5)	12.6±7.3	7.52±0.81	103 (8.5)	24.2±26.1
Moderate or severe renal damage	80.1±7.8	1,392 (58.0)	12.2±7.4	7.60±0.75	266 (11.0)	24.8±24.0
Leukemia	80.5±7.5	95 (56.2)	13.2±7.9	7.27±1.21	30 (17.6)	22.3±20.8
Moderate or severe liver disease	77.8±7.5	101 (54.6)	13.0±8.3	7.42±1.1	30 (16.2)	23.9±38.7
Metastatic solid tumor	79.0±7.5	449 (56.4)	12.2±8.0	7.53±0.84	119 (14.9	23.5±24.0

Table 7 summarizes the associations between pre-existing comorbidities and post-admission complications significantly associated with in-hospital mortality, as assessed using Fisher’s exact test. Congestive heart failure was significantly associated with cardiovascular complications, acute renal failure, pneumonia, gastrointestinal complications, sepsis, and infection (all p<0.05). Chronic lung disease was significantly associated with cardiovascular complications, acute renal failure, pneumonia, gastrointestinal complications, and infection. Mild liver disease was significantly associated with central nervous system complications, cardiovascular complications, acute renal failure, pneumonia, gastrointestinal complications, genitourinary complications, DIC, and infection, indicating broad involvement across multiple complication types. Moderate or severe renal impairment was significantly associated with cardiovascular complications, acute renal failure, pneumonia, gastrointestinal complications, and sepsis. Leukemia was significantly associated with central nervous system complications and pneumonia. Moderate or severe liver disease was significantly associated with abdominal compartment syndrome, gastrointestinal complications, and sepsis. Metastatic solid tumors were significantly associated with cardiovascular complications and pneumonia. In contrast, connective tissue disease was significantly associated only with central nervous system and genitourinary complications.

**Table 7:** Association between pre-injury comorbidities and post-admission complications (n=77,306) p-value calculated by Fisher's exact probability test. *Statistically significant; p<0.05. CNS: Central nervous system.

	CNS complications	Cardio-vascular complications	Acute renal failure	Abdominal compartment syndrome	Pneumonia	Digestive system disorders	Genitourinary complications	Disseminated intravascular coagulation	Sepsis	Infectious disease
	(n=3,069)	(n=1,678)	(n=333)	(n=17)	(n=4,202)	(n=1,387)	(n=14,010)	(n=251)	(n=277)	(n=3,782)
Congestive heart failure (n=3,129)	0.784	<0.001*	<0.001*	0.505	<0.001*	0.011*	0.76	0.2	0.046*	<0.001*
Chronic pulmonary disease (n=2,806)	0.994	<0.001*	0.017*	1	<0.001*	0.006*	0.519	0.395	0.332	<0.001*
Connective tissue disease (n=1,099)	0.007*	0.987	0.639	0.216	0.078	0.208	0.049*	0.785	0.801	0.396
Mild liver disease (n=1,208)	0.001*	<0.001*	0.007*	1	0.003*	<0.001*	0.018*	0.018*	0.335	0.034*
Moderate or severe renal damage (n=2,414)	0.069	<0.001*	0.006*	0.417	<0.001*	<0.001*	0.38	0.366	0.036*	0.47
Leukemia (n=170)	0.047*	0.102	0.167	1	0.016*	1	0.775	1	0.125	0.722
Moderate or severe liver disease(n=185)	0.055	0.067	0.55	<0.001*	0.514	<0.001*	0.735	0.452	0.029*	0.492
Metastatic solid tumor (n=800)	0.273	0.007*	0.091	1	0.002*	0.139	0.607	0.748	1	0.324

## Discussion

Older patients frequently have pre-existing or concomitant conditions that may influence in-hospital outcomes following trauma injury [20]. Therefore, even when injury severity appears lower than that observed in younger trauma patients, it is reasonable to consider background factors - including pre-existing or concomitant conditions and associated medications - when determining the appropriate destination hospital for older trauma patients. The JTDB, a nationwide trauma registry in Japan, includes information on 19 comorbidity items based on the CCI, which was not originally developed for trauma-specific risk assessment. We previously analyzed the association between the pre-existing and concomitant conditions and in-hospital mortality in blunt trauma patients using JTDB data from 2019 to 2022, without age restriction [20]. In the present study, using JTDB data from 2019 to 2023, we focused specifically on older trauma patients and included both blunt and penetrating injuries to examine the association between individual pre-existing comorbidities and in-hospital mortality. Notably, the CCI components significantly associated with in-hospital mortality were consistent with those identified in the previous report [20], suggesting that eight specific pre-existing conditions are particularly relevant to trauma prognosis.

Comparisons between the in-hospital mortality and survival groups (Tables 1, 2) demonstrated significant differences in age, sex, RTS, and ISS. Multivariate Cox proportional hazards analysis further showed that age remained independently associated with mortality risk, even within a cohort restricted to older patients. This finding supports the concept that age-related declines in physiological reserve may influence prognosis independently of injury severity. The CCI items significantly associated with mortality - leukemia, moderate or severe liver disease, metastatic malignancy, moderate or severe renal disease, congestive heart failure, connective tissue disease, chronic lung disease, and mild liver disease - were consistent with those reported previously [20]. Importantly, even when the analyses were limited to patients aged ≥65 years and included both blunt and penetrating trauma, the same comorbidities remained significant predictors of in-hospital mortality. Although mild liver disease showed a significant association at baseline in the previous study, this association was attenuated after bootstrap adjustment, likely reflecting the increased sample size and restriction to an older population. Collectively, these findings indicate that among the 19 original CCI components [4], these eight conditions warrant particular attention in trauma care.

Hierarchical cluster analysis using Ward’s method (Table 6 and Figure 2) showed that moderate or severe liver disease, metastatic solid tumors, leukemia, and moderate or severe renal disease formed closely related clusters, whereas chronic lung disease, mild liver disease, and congestive heart failure constituted a separate cluster. In contrast, connective tissue disease remained isolated from other comorbidities. Analysis of associations between pre-existing comorbidities and post-admission complications (Table 4) showed that most comorbidities were significantly associated with organ-specific complications after admission, whereas connective tissue disease was significantly associated only with central nervous and urinary system complications. Most comorbidities show broad, non-specific associations across multiple complication categories, while connective tissue disease showed a comparatively narrower pattern. This pattern suggests a post-admission course distinct from that observed with other comorbidities. Older trauma patients with conditions, such as leukemia, moderate or severe liver disease, metastatic malignancy, and connective tissue disease, may share underlying factors that impair host defense against infection. Even when acute circulatory failure due to hemorrhage is successfully managed, these patients may develop wound-related infections during the subacute or later phases, potentially leading to sepsis or organ failure [21,22].

Connective tissue diseases were significantly isolated from other pre-existing comorbidities and were more prevalent among women. It was significantly associated only with central nervous system and genitourinary complications. However, treatments, such as long-term steroid use, may impair immune function and increase susceptibility to infection, necessitating careful clinical consideration. Furthermore, comparison of admission data between patients with and without a history of connective tissue disease showed that, despite lower injury severity and fewer severe head injuries, patients with connective tissue disease had a significantly lower rate of favorable neurological outcomes (data not shown). Older trauma patients with connective tissue disease who are currently receiving oral steroids, or who have a history of long-term steroid use, may therefore experience poorer neurological outcomes following severe head injury. In such cases, the decision of the receiving hospital might not rely solely on conventional trauma severity classification, and the risk of undertriage should be carefully avoided. Further investigation focusing specifically on connective tissue disease as a pre-existing condition might provide additional insights relevant to emergency medicine. It will require further consideration.

Limitation

This study has some limitations. First, as a retrospective analysis using the JTDB, some variables contained missing data, and cases lacking required information were excluded from analyses as appropriate. Second, Japan’s prehospital emergency transport system has unique characteristics, including its paramedic framework [23], which may limit the generalizability of these findings to other healthcare systems. Third, the relatively low prevalence of certain comorbidities may have reduced statistical power, indicating the need for validation using other large-scale datasets. Fourth, in the exploration of clustering and complication analysis, information was missing regarding misclassification risk, lack of treatment drug data, and inadequate cause-of-death classification. Fifth, the methods for identifying complications vary between hospitals, potentially introducing bias by affecting interpretation.

## Conclusions

This study revealed that in-hospital mortality among older trauma patients is associated not only with injury severity but also with specific pre-existing comorbidities. Pre-existing cardiac, pulmonary, hepatic, or renal dysfunction, as well as malignant disease, were independent risk factors for mortality in older patients with severe trauma and were associated with the development of various post-admission complications. Among these comorbidities, connective tissue disease was associated with a unique pattern of complications following hospitalization, suggesting a different occurrence of complications compared with other pre-existing conditions in older trauma patients.

## References

[1] Atinga A, Shekkeris A, Fertleman M, Batrick N, Kashef E, Dick E (2018). Trauma in the elderly patient. Br J Radiol.

[2] Gauss T, de Jongh M, Maegele M, Cole E, Bouzat P (2024). Trauma systems in high socioeconomic index countries in 2050. Crit Care.

[3] Chua MT, Pan DS, Lee MZ (2023). Comparing Comorbidity Polypharmacy Score and Charlson Comorbidity Index in predicting outcomes in older trauma patients. Injury.

[4] Charlson ME, Pompei P, Ales KL, MacKenzie CR (1987). A new method of classifying prognostic comorbidity in longitudinal studies: development and validation. J Chronic Dis.

[5] Cusimano MD, Saarela O, Hart K, Zhang S, McFaull SR (2020). A population-based study of fall-related traumatic brain injury identified in older adults in hospital emergency departments. Neurosurg Focus.

[6] Parreira JG, Farrath S, Soldá SC, Perlingeiro JA, Assef JC (2013). Comparative analysis of trauma characteristics between elderly and superelderly. Rev Col Bras Cir.

[7] Baker SP, O'Neill B, Haddon W Jr, Long WB (1974). The injury severity score: a method for describing patients with multiple injuries and evaluating emergency care. J Trauma.

[8] Champion HR, Sacco WJ, Copes WS, Gann DS, Gennarelli TA, Flanagan ME (1989). A revision of the Trauma Score. J Trauma.

[9] Champion HR, Copes WS, Sacco WJ (1990). The Major Trauma Outcome Study: establishing national norms for trauma care. J Trauma.

[10] Savioli G, Ceresa IF, Ciceri L (2020). Mild head trauma in elderly patients: experience of an emergency department. Heliyon.

[11] Kishawi SK, Mahajan A, Nahmias J, Petrone P, Ho VP (2025). Patterns and implications of missed injuries on computed tomography imaging in older blunt trauma patients. Surgery.

[12] Jarman MP, Jin G, Weissman JS (2022). Association of trauma center designation with postdischarge survival among older adults with injuries. JAMA Netw Open.

[13] Kudu E, Altun M, Danış F, Karacabey S, Sanri E, Denizbasi A (2025). Validating the falls decision rule: optimizing head CT use in older adults with ground-level falls. CJEM.

[14] Abe T, Komori A, Shiraishi A, Sugiyama T, Iriyama H, Kainoh T, Saitoh D (2020). Trauma complications and in-hospital mortality: failure-to-rescue. Crit Care.

[15] Kainoh T, Iriyama H, Komori A, Saitoh D, Naito T, Abe T (2021). Risk factors of fat embolism syndrome after trauma: a nested case-control study with the use of a nationwide trauma registry in Japan. Chest.

[16] Japan Trauma Data Bank. https://www.jtcr-jatec.org/traumabank/index.htm.

[17] Yokota J (2016). Japan Trauma Data Bank (JTDB) managed by Japan Trauma Care and Research (JTCR) (in Japanese). Nihon Rinsho.

[18] Teasdale G, Maas A, Lecky F, Manley G, Stocchetti N, Murray G (2014). The Glasgow Coma Scale at 40 years: standing the test of time. Lancet Neurol.

[19] Abbreviated Injury Scale. Association for the Advancement of Automotive Medicine. Abbreviated Injury Scale.

[20] Noguchi S, Saitoh D, Tanaka H (2025). Association between Charlson Comorbidity Index items and outcomes in patients with blunt trauma using a nationwide trauma registry database in Japan. Cureus.

[21] Hassoun HT, Kone BC, Mercer DW, Moody FG, Weisbrodt NW, Moore FA (2001). Post-injury multiple organ failure: the role of the gut. Shock.

[22] Probst C, Zelle BA, Sittaro NA, Lohse R, Krettek C, Pape HC (2009). Late death after multiple severe trauma: when does it occur and what are the causes?. J Trauma.

[23] Mishima H, Nakagawa K, Takeuchi H (2024). Impact of pre-hospital intravenous infusion on physiological parameters in severe trauma patients. Cureus.

